# False Seedbed and Stale Seedbed Against Important Broadleaf Weeds: A Case Study and a Step Closer to Agroecology

**DOI:** 10.3390/plants14040564

**Published:** 2025-02-12

**Authors:** Panagiotis Kanatas, Ioannis Gazoulis, Dimitra Petraki, Metaxia Kokkini, Nikolaos Antonopoulos, Ilias Travlos

**Affiliations:** 1 Department of Crop Science, University of Patras, 30200 Mesolonghi, Greece; pakanatas@upatras.gr (P.K.); giangazoulis@gmail.com (I.G.); 2 Department of Crop Science, Agricultural University of Athens, 75, Iera Odos Str., 11855 Athens, Greece; dimpetraki9@gmail.com (D.P.); kokinimetaxia2000@gmail.com (M.K.); nikolasantwno@gmail.com (N.A.)

**Keywords:** false seedbed, stale seedbed, wheat, weed management

## Abstract

Wheat production can be reduced due to competition from weeds, with farmers relying almost exclusively on chemical solutions. However, there are alternative farming practices available. Therefore, in a field trial in Greece, we assessed the efficacy of false and stale seedbed against important broadleaf weed species and their impact on grain yield parameters. Our study determined that false seedbed resulted in a decrease in the density and biomass of broadleaf weed species like catchweed bedstraw (*Galium aparine* L.) up to 75% and 69% compared with the untreated control (normal seedbed preparation), respectively. The efficacy of false seedbed was higher when combined with post-emergence chemical control. Stale seedbed also resulted in adequate weed control, with a biomass reduction of up to 81%, and grain yield increased by 30% compared with the normal seedbed preparation without any herbicide use. Our results highlight the differences in the response between the weed species and also emphasize the potential of adding an alternative farming practice (like false and stale seedbed) as part of an integrated farming strategy for a sustainable and agroecological crop and weed management. In order to validate the trends observed in this case study, further field-to-field or year-to-year replication is required.

## 1. Introduction

Weed competition can be a major obstacle limiting grain yields in the most important crops like wheat (23%), rice (37%), and maize (40%) [1,2]. Weed management usually relies on herbicides, partially due to their relatively easy, cheap, and fast application [3,4]. However, this over-reliance on chemical control resulted in the evolution of herbicide-resistant weed biotypes in both annual and perennial crops [5,6]. Currently, there is a focus on using non-herbicide methods to manage weeds due to several concerns about the non-target impacts of these chemicals [7,8].

False and stale seedbeds are formed when the field is prepared for sowing, and weeds are provoked to germinate after initial tillage operations and later destroyed by means of a shallow tillage operation or herbicide application, respectively [9,10]. However, the efficacy of such farming practices on weed control varies widely based on many factors, including the crop, the weed community, the nutrients, the tillage, and the specific soil and climatic conditions [11,12,13,14]. For instance, false seedbed in barley reduced the biomass of weed by up to 64% compared with direct sowing and normal seedbed preparation [9]. The application of this specific method in crops like rice, soybean, and cotton also reduced weed density and increased crop yield [15,16]. Despite these potential benefits, there are cases like in compact soils with heavy rainfall in which false seedbed can significantly delay crop establishment and put crop productivity at risk [17].

This study evaluates the performance of false and stale seedbed in winter wheat grown in central Greece. The main objective of this study was to assess the farming practices of false and stale seedbed and their combinations compared with normal seedbed preparation against important broadleaf weeds and the productivity of wheat crop.

## 2. Results

**Figure 1:**
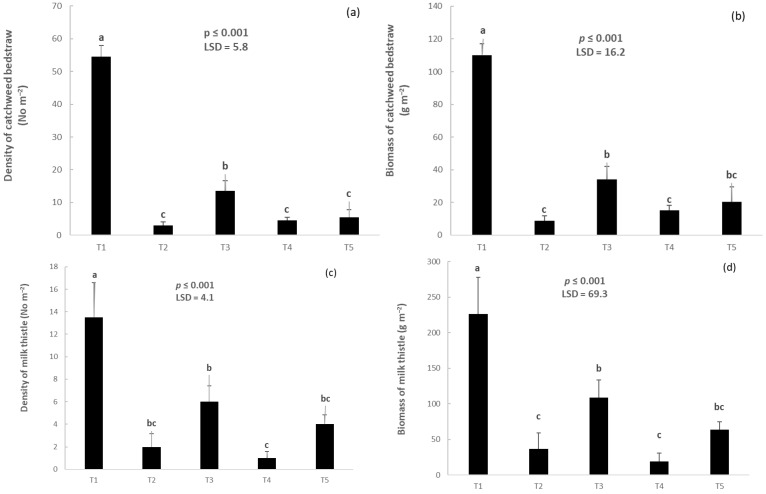
Density of catchweed bedstraw (**a**), biomass of catchweed bedstraw (**b**), density of milk thistle (**c**), and biomass of milk thistle (**d**). Vertical bars denote standard errors of the means, and different lowercase letters indicate statistically significant differences between the treatments (according to Fisher’s LSD test) (T1: normal seedbed, T2: normal seedbed followed by early post-emergence herbicide application, T3: false seedbed, T4: false seedbed followed by early post-emergence herbicide application, T5: stale seedbed).

False seedbed (T3) reduced the density of catchweed bedstraw by 75% compared with the normal seedbed preparation (T1). The density of this weed varied between 1.0 and 5.5 plants m^−2^ in the plots where the other weed control methods were applied (Figure 1a). Treatments T3 (false seedbed) and T5 (stale seedbed) reduced the biomass of catchweed bedstraw by 69 and 81% compared with T1. T4 (false seedbed followed by early post-emergence herbicide application) resulted in similar values as T5 and T2 (Figure 1b). Milk thistle (*Silybum marianum* (L.) Gaertn.) density was highest in the untreated plots (T1) and lowest in T4 (Figure 1c). Intermediate densities of this weed corresponded to treatments T3 and T5. Even lower densities were recorded in T2 (2.0 plants m^−2^). Milk thistle biomass decreased to 108.6 g m^−2^ in the T3 and T5 plots. T2 and T4 reduced milk thistle biomass by 83 and 92%, respectively, compared with T1 (Figure 1d).

**Figure 2:**
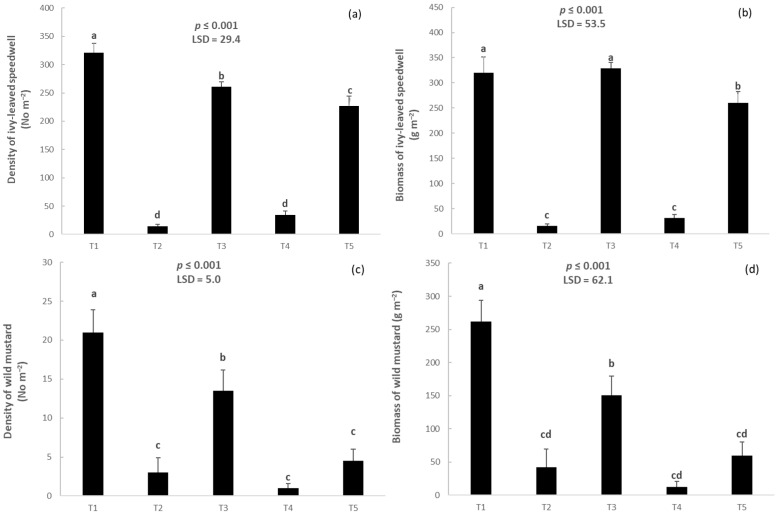
Density of ivy-leaved speedwell (**a**), biomass of ivy-leaved speedwell (**b**), density of wild mustard (**c**), and biomass of wild mustard (**d**). Vertical bars denote standard errors of the means, and different lowercase letters indicate statistically significant differences between the treatments (according to Fisher’s LSD test) (T1: normal seedbed, T2: normal seedbed followed by early post-emergence herbicide application, T3: false seedbed, T4: false seedbed followed by early post-emergence herbicide application, T5: stale seedbed).

The density of ivy-leaved speedwell (*Veronica hederifolia* L.) in the T1 plots was higher than 300 plants m^−2^. Treatment T3 reduced the density of ivy-leaved speedwell by only 16% compared with T1. T5 was more effective than T3 but still had high density. On the contrary, T2 and T4 resulted in the lowest densities of this weed (Figure 2a). The dry weight of ivy-leaved speedwell per unit area did not differ between T1 and T3, while T5 had a significantly lower biomass of this weed (but still high as shown in Figure 2b). T2 and T4 resulted in a very good control (≥90%) of ivy-leaved speedwell. A similar trend was observed for wild mustard (*Sinapis arvensis* L.); T4 almost eliminated this species from the respective experimental plots (Figure 2c). T3 reduced weed density by 36% compared with T1. In addition, T5 resulted in a lower weed density than T3. T2 and T4 were the most effective treatments in terms of weed control. The biomass of wild mustard followed a descending order: T1 > T3 > T5 ≥ T2 ≥ T4 (Figure 2d).

**Figure 3:**
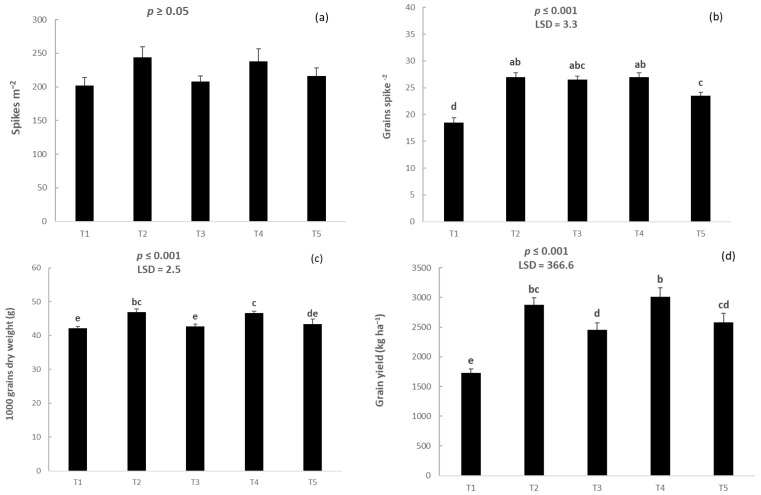
(**a**) The number of spikes per unit area (spikes m^−2^), (**b**) the number of grains per spike (grains spike^−1^), (**c**) the weight of 1000 grains (g), and (**d**) the final grain yield of durum wheat (kg ha^−1^). Vertical bars denote standard errors of the means, and different lowercase letters indicate statistically significant differences between the treatments (according to Fisher’s LSD test) (T1: normal seedbed, T2: normal seedbed followed by early post-emergence herbicide application, T3: false seedbed, T4: false seedbed followed by early post-emergence herbicide application, T5: stale seedbed).

Regarding the first measured component of grain yield, the number of spikes per unit area was not affected by the treatment (*p* ≥ 0.05; Figure 3a). T5 produced 23.5 grains spike^−1^, which was lower compared with the corresponding values of T2 and T4 but higher than the corresponding value of the untreated control (Figure 3b). The weight of 1000 grains was the highest in plots T2 and T4, while it was the lowest in plots T5, T3, and T1 (Figure 3c). T4 increased grain yield by 43% compared with the untreated control. Furthermore, false seedbed without any chemical control resulted in a grain yield increase of 30% compared with the normal seedbed preparation without any herbicide use (Figure 3d).

## 3. Discussion

**Figure 4:**
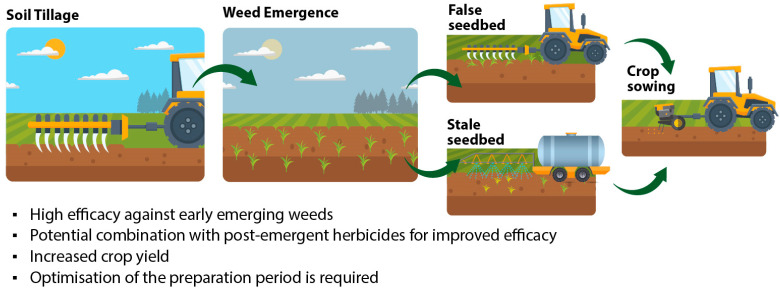
Key findings of the present study.

The results of this study indicate that false and stale seedbeds give growers the ability to control early-emergent weed cohorts (Figure 4). Although this sounds promising, later-emergent weeds may not be controlled and interfere with the crop later in the season. This was the case with ivy-leaved speedwell and wild mustard, in which false and stale seedbeds had low efficacy. Regarding the whole weed flora, false and stale seedbeds reduced the total weed biomass by 33 and 56%, respectively, compared with the normal seedbed preparation. This is in accordance with previous studies [18,19] and could be adopted not only in order to reduce weed pressure and competition to the crops but also to favor less competitive or even some “service weeds”, as proposed by Gazoulis et al. [20].

Our results also determine that after weed control by shallow tillage in false seedbed or application of non-selective herbicides in stale seedbed, selective post-emergence herbicide applications are often required to achieve optimal weed control and higher crop yield, which is in accordance with previous studies [9]. However, it should always be noted that false and stale seedbeds control the first weed flushes, causing a depletion of the weed seedbank in the soil, which can reduce weed pressure at a given field in the long term, as previously discussed by Merfield et al. [11]. In addition, false and stale seedbeds may be even more necessary in crops where there are not so many available herbicides that are registered for weed control [19,21].

Another important observation of our study was that stale seedbeds tended in general to be more effective than false seedbeds, and this agrees with recent previous research [12,21]. This could be due to the fact that even shallow tillage operations cause some soil disturbance, bringing a few more weed seeds to the upper soil layers where they can rapidly germinate [22,23]. However, the efficacy of such an approach is not something absolute and consistent for all weed species (e.g., some grass species could be tolerant); therefore, its application along with other methods for the rapid evaluation of herbicide efficacy under real field conditions is recommended [10]. In addition, weed species may have variable sensitivity to false and stale seedbed preparation due to their seed size, earliness, and thermal requirements [24]. For instance, in our study, false and stale seedbeds were more efficient against *G. aparine* than *V. hederifolia*.

The optimal period of time in which sowing is delayed between the initial seedbed preparation and control of the germinated weeds should be defined because this could be one of the main reasons for the various efficacy levels [25]. It can be hypothesized that the more days sowing is delayed, the more seedlings will germinate and be subsequently controlled. However, extended periods of delay may result in yield losses at the end of the growing season because the biological cycle of the crop may be significantly shortened. Also, previous studies have shown that delayed sowing dates can reduce the use intensity of herbicides with a high resistance risk [26]. In any case, such questions on the optimization of the above-mentioned farming practices and their interaction with other methods should be addressed with more field trials because results may be dependent on the soil and climatic conditions prevailing at a given agricultural site.

## 4. Materials and Methods

A field trial was conducted during the 2023–2024 growing season in a durum wheat field in a typical cereal-growing area of central Greece (Velestino area, 39.104° N, 22.293° E). Durum wheat was the previous crop (typical monoculture). Catchweed bedstraw, milk thistle, ivy-leaved speedwell, and wild mustard were the dominant weeds. Soil type was clay (C) with a pH of 7.32 and an organic matter content of 2.59%. The climatic conditions that prevailed in the experimental field from October 2023 until June 2024 are shown in Table 1.

The soil was plowed to a depth of about 30 cm in early October and then cultivated with a cultivator to a depth of 20 cm for seedbed preparation and fertilizer incorporation. A complete inorganic fertilizer 18-23-0 was incorporated into the soil at a rate of 250 kg ha^−1^ to provide the crop with 45 kg N ha^−1^ and 57.5 kg P_2_O_5_ ha^−1^. The durum wheat cultivar “Maesta” was used with row spacing of 15 cm and a seeding rate of 260 kg ha^−1^.

A randomized complete block design (RCBD) with five treatments and four replications (blocks) was established. The treatments included normal seedbed (T1), normal seedbed followed by early post-emergence herbicide application (Τ2), false seedbed (Τ3), false seedbed followed by early post-emergence herbicide application (Τ4), and stale seedbed with glyphosate at 720 g a.e. ha^−1^ (T5). Plots were 4 m long and 5 m wide, resulting in a plot area of 20 m^2^. The whole experimental area was 400 m^2^. Weed-free borders (40 cm) were kept between adjacent plots. The term “normal seedbed” refers to the conventional practice of sowing the crop (with a hand seeder) the day after seedbed preparation. The exact date of sowing in T1 and T2 plots was 29 November 2023.

**Figure 5:**
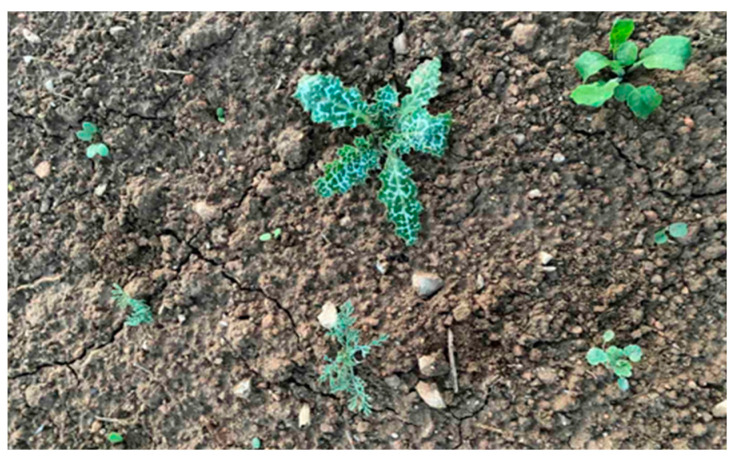
Emerged weed seedlings before false and stale seedbed completion.

The term “false seedbed” means that sowing was delayed for around two weeks after seedbed preparation (Figure 5). In the meantime, weed seedlings were left to germinate and grow in T3 and T4 plots. Before sowing, the emerged weeds were controlled with a shallow tillage operation (with a handheld gasoline-powered rototiller) at a depth of around 10 cm. Right after weed control with shallow tillage, sowing was carried out on 13 December 2023. The term “stale seedbed” also means that sowing was delayed for around two weeks and that the emerged weed seedlings were controlled before sowing by glyphosate (Roundup Flex^®^, Bayer Hellas S.A., Athens, Greece) at 720 g a.e. ha^−1^ in T5.

For the early post-emergence herbicide applications in T2 and T4 plots, diflufenican + flufenacet + metribuzin (Herold Trio^®^, Bayer Hellas S.A., Athens, Greece) was used. The application rate of the commercial herbicide product was 700 cc ha^−1^, with the rates for diflufenican, flufenacet, and metribuzin being 119.7, 119.7, and 445.7 g a.i. ha^−1^, respectively. Durum wheat was at the three-leaf growth stage (Zadoks 13), and the exact date of the application (in both fields) was 23 January 2023. Herbicides were applied using a “Volpi V. black Elektron” battery sprayer (Davide & Luigi Volpi S.p.a., Mantua, Italy) calibrated to deliver 300 L ha^–1^ of spray solution through a brass conical nozzle at a constant pressure of 200 kPa for glyphosate and 300 kPa for the diflufenican + flufenacet + metribuzin.

Weed density was estimated on 27 March 2024. To conduct measurements, two metallic quadrats with a size of 0.25 m^2^ were placed in each plot in areas with uniform weed flora and away from the margins. Weed counts were performed to compare treatments and estimate their efficacy on the dominant weed species. In addition, weed biomass was assessed on the same day. The weeds were harvested with scissors at the ground level, classified by species, packed in numbered plastic bags, and taken to the Laboratory of Agronomy of the Agricultural University of Athens. The weed samples were then oven-dried at 65 °C for 48 h, and the weed biomass was measured using a digital scale. The crop was harvested on 23 June 2024. From each plot, spikes were taken from a central 1 m^2^ area delimited by a wooden square, and the samples were placed in numbered plastic bags. The number of spikes per unit area and the number of grains per spike were measured by taking a representative sample of 50 spikes from each plot. The weight of 1000 grains was also measured for each plot. After multiplying these three yield components, the final grain yield of durum wheat was estimated.

All data were subjected to one-way Analysis of Variance (ANOVA). Treatments were considered fixed effects, and replications were the random effects at a significance level of *a* = 0.05 (df_Treatment_ = 4). Multiple comparisons between treatments’ means were conducted using Fischer’s Least Significant Difference (LSD) test. Statgraphics Centurion XVI (Statgraphics Technologies, Inc., P.O. Box 134, The Plains, VA, USA) was the statistical package used for all data analyses.

## 5. Conclusions

Our study determined that false seedbeds resulted in a decrease in the density of broadleaf weed species by up to 75% compared with the untreated control, while the efficacy was higher when combined with post-emergence chemical control. Stale seedbeds also resulted in adequate weed control, with a biomass reduction of up to 81% and a grain yield increase of 30% compared with the normal seedbed preparation without any herbicide use. Our study highlights the different responses of the weed species due to their different biological cycle and also emphasizes the crucial role of farming practices such as false and stale seedbeds in sustainable and agroecological crop and weed management. In all cases, further field-to-field or year-to-year replication is required. Also, for the validation of the trends revealed in this case study, further experimentation ought to be conducted under various pedoclimatic conditions.

## Figures and Tables

**Table 1:** Average, maximum, and minimum temperature (C) and precipitation (mm) during the wheat cycle in the experimental area.

Year	Month	Average Temperature (°C)	Maximum Temperature (°C)	Minimum Temperature (°C)	Monthly Precipitation (mm)
2023	October	16.2	25.3	9.6	105.1
	November	11.2	19.4	5.4	54.2
	December	7.3	13.4	2.4	46.6
2024	January	6.2	11.7	0.6	31.6
	February	7.1	13.6	1.5	40.0
	March	10.1	15.2	3.1	38.2
	April	13.1	21.1	6.6	29.0
	May	18.8	26.3	9.8	35.2
	June	24.3	31.3	14.6	24.0

## Data Availability

Data is contained within the article.
